# A Systematic Review of Factors Critical for HIV Health Literacy, ART Adherence and Retention in Care in the U.S. for Racial and Ethnic Minorities

**DOI:** 10.1007/s10461-022-03680-y

**Published:** 2022-04-21

**Authors:** Ofole Mgbako, Ryan Conard, Claude A. Mellins, Jagadisa-devasri Dacus, Robert H. Remien

**Affiliations:** 1 Division of Infectious Diseases, Department of Medicine, NYU Langone Health, New York, NY USA; 2 NYU Institute for Excellence in Health Equity, NYU Langone Health, New York, NY USA; 3 HIV Center for Clinical and Behavioral Studies, NY State Psychiatric Institute and Columbia University, New York, NY USA; 4 Program in Liberal Medical Education, Brown University, Providence, Rhode Island, USA; 5 Institute for Sexual and Gender Minority Health and Wellbeing, Northwestern University, Chicago, IL USA; 6 300 Ashland Place, Apt 19B, Brooklyn, NY 11217 USA

**Keywords:** HIV, Health literacy, ART, Adherence, Retention in care

## Abstract

Despite advances in antiretroviral treatment (ART), the HIV epidemic persists in the United States (U.S.), with inadequate adherence to treatment and care a major barrier to ending the epidemic. Health literacy is a critical factor in maximizing ART adherence and healthcare utilization, especially among vulnerable populations, including racial and ethnic minorities. This U.S-based systematic review examines psychosocial variables influencing health literacy among persons with HIV (PWH), with a focus on racial and ethnic minorities. Although findings are limited, some studies showed that HIV-related stigma, self-efficacy, and patient trust in providers mediate the relationship between health literacy and both ART adherence and HIV care retention. To inform effective, equitable health literacy interventions to promote adherence to HIV treatment and care, further research is needed to understand the factors driving the relationship between health literacy and HIV outcomes. Such work may broaden our understanding of health literacy in the context of racial equity.

## Introduction

Despite significant advances in effective antiretroviral treatment (ART), the HIV epidemic persists in the United States (U.S.) in part due to insufficient ART adherence and low retention in care among persons with HIV (PWH). In 2018, only 50% of PWH in the U.S. were retained in care [1]. Success along the HIV care continuum requires some degree of health literacy for PWH to understand how HIV affects the body, to initiate and adhere to specific ART regimens in order to achieve ongoing viral suppression, and to navigate ongoing HIV care [2, 3]. Health literacy is defined as one's ability to acquire, communicate and manage basic health information and services to make informed health decisions [4]. Health literacy, in this way, differs from basic medical knowledge. It encompasses not only basic understanding of health issues, but also the capacity to grasp complex medical concepts and make well-informed health decisions [4]. Previous systematic reviews of health literacy and HIV care have focused on the link between health literacy, HIV risk behaviors and HIV care outcomes, or the range and effects of different health literacy interventions [5–8]. Yet few have explored the psychosocial constructs that shape health literacy among PWH. Such information could inform evidence-based interventions to promote health literacy, as well as adherence and retention, which are currently limited.

Health literacy itself is a complex construct [9]. Studies of the impact of health literacy on HIV-related health behaviors have often been limited by the use of a) cross-sectional study designs, limiting knowledge of causal priority, and b) a variety of different measures [Test of Functional Health Literacy in Adults (TOFHLA); Rapid Estimate of Adult Literacy in Medicine (REALM)] precluding cross-study comparisons [10, 11]. Furthermore, although previous systematic reviews have identified studies that focused on the association between health literacy and demographic factors such as race and ethnicity, a nuanced, holistic understanding of *how* health literacy impacts HIV-related health behaviors for groups with the highest HIV incidence is missing from the literature. It is vital to understand the other variables in the causal relationship between health literacy and HIV outcomes to inform acceptable, accessible, and effective interventions.

It is well known that racial and ethnic minorities bear the disproportionate burden of the HIV epidemic, with 42% of Black and 27% of Latinx Americans making up new HIV diagnoses in the U.S. in 2018 [12]. Low health literacy has been identified as a potential driver of both HIV acquisition and subsequent poor HIV care outcomes among racial and ethnic minorities [13, 14]. Yet one’s level of health literacy is not static, nor does it reflect an intrinsic characteristic of the individual. More generally, current health literacy literature in HIV lacks a critical focus on the psychosocial variables that mediate the relationship between race, health literacy and HIV outcomes. One can imagine even patients with high health literacy may have poor ART adherence or retention in care due to a range of factors, such as a lack of social support or isolation and lack of diagnosis disclosure, or the role of HIV care systems and/or providers failing to establish welcoming care environments in a rapidly changing HIV care landscape.

A broader understanding of the complexity of health literacy’s influence in HIV care is necessary to promote HIV adherence and retention outcomes and reduce racial and ethnic disparities among vulnerable PWH. We conducted a systematic review to assess dynamic contextual factors that inform a patient’s ability to comprehend and process health-related information as they interact with the HIV care system. By attending to the range of psychosocial variables that mediate or moderate the association between health literacy and retention in HIV care, and the association between health literacy and ART adherence identified in existing literature, this review contributes a more comprehensive picture of health literacy. We focused our review specifically in the U.S given the state of the national HIV epidemic, that HIV healthcare delivery systems vary substantially country by country, and that health literacy is informed by context-specific norms, language, and culture.

## Methods

An electronic literature search was carried out in PubMed, Google Scholar, Embase, and CINAHL during June 2020. Search terms included a combination of “health literacy” or “literacy” and any of the following: “HIV care,” “retention in HIV care,” “adherence to ART,” “adherence to HIV medications,” “HIV visit adherence,” and “HIV treatment adherence.” PRISMA guidelines were used to find and narrow down the list of studies [15]. Titles, abstracts, and study methods were initially reviewed. Studies were included in the review if they examined at least in part, the relationship between health literacy and 1) ART adherence, or 2) retention in care. Only studies written in English were included.

Studies using either quantitative or qualitative research methods were included that were published after 1996, the year of the advent of highly active antiretroviral treatment (HAART), in order to eliminate suboptimal ART regimens as a reason for poor ART adherence. Quantitative studies were excluded if they focused exclusively on one health literacy intervention or if they did not use a validated health literacy measuring tool, which we defined as a tool that has been studied in the literature for its accuracy, validity, reliability, and effectiveness in measuring health literacy.

Intervention studies were specifically excluded because they presumed an association between health literacy and medication adherence or retention in care. In seeking to identify important psychosocial variables associated with health literacy, we sought to identify only studies that explicitly examined whether an association between health literacy and HIV outcomes existed, and any psychosocial variables that might impact that relationship. Additionally, health literacy intervention studies often involve multiple components and are quite heterogeneous, with interventions involving varied personnel, structure, approach, setting, and population. Given the diversity of health literacy interventions in different studies, it would be difficult to identify which aspects of the intervention led to a desired effect in HIV care outcomes.

Quantitative studies were also excluded if they did not use one of the commonly cited behavioral measures of adherence (e.g., self-report, pill counts) or retention in care (e.g., proportion of scheduled appointments actually attended; HRSA definition of greater than two visits in one year at least 90 days apart) [16–18]. Qualitative studies were excluded if study participants were not specifically asked about health literacy in HIV care as part of a focus group or in an in-depth interview.

## Results

**Fig. 1:**
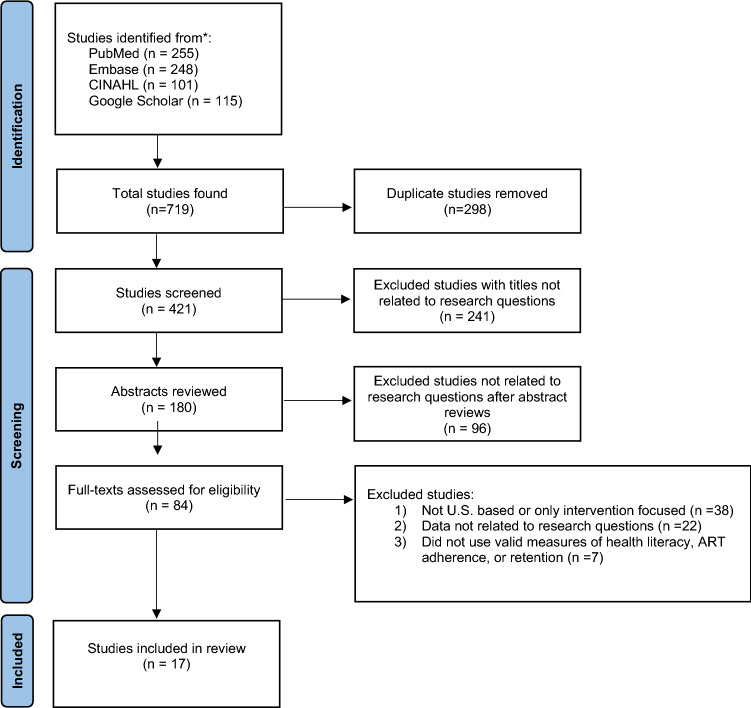
PRISMA flow diagram of systematic review

Our search of the various databases identified 719 studies. After removing duplicates, the remaining 421 titles were screened for relevance. Of those, 241 titles were excluded, given that they did not examine the association between health literacy and HIV outcomes. Among the remaining 180 abstracts, we identified an additional 96 that did not examine the association between health literacy and HIV outcomes. Thus, 84 full texts were assessed for eligibility. Of these, 17 studies, 14 quantitative and 3 qualitative, met the full criteria for inclusion in this review as the remainder were not related to the research question, or were not U.S.-based. There were a range of measures and/or scales used to assess health literacy, ART adherence, and retention in care. All 17 studies addressed whether health literacy was associated with retention in HIV care and/or ART adherence. Figure 1 illustrates a flow chart of how the studies included were found, sorted, and chosen. Table 1 contains a comprehensive summary of the studies.

**Table 1:** Summary of Studies Identified and Used for this Review

Reference	Authors	Study Population	Health Literacy/ Adherence/ Retention Measures	Association between Health Literacy and Retention in Care?	Association between Health Literacy and ART Adherence?	Other Psychosocial Factors	Limitations
*Quantitative articles*							
Health Literacy and Adherence to Antiretroviral Therapy Among HIV-Infected Youth (2014)	Navarra et al	50 adolescents (ages 13–24 years)	Health literacy measured via TOFHLA and REALM-teen Adherence measured through a 3-day self-report of missed doses		Health literacy was not predictive of adherence (OR = 0.954, 95%CI 0.893–1.018; p = 0.15)	Higher positive outcome expectancy led to increased adherence (OR = 1.07, 95%CI 1.02–1.12; p = 0.006)	Small sample size; self-reported data; wide age range of participants; style of surveys may have influenced responses
Health Literacy and Antiretroviral Adherence Among HIV-infected Adolescents (2010)	Murphy et al	186 adolescents (ages 16–24) from five US cities	Health literacy measured via TOFHLA Adherence measured via Pediatric Adherence Questionnaire (missed dosages over 3 days)		No significant association between medication adherence and health literacy (OR = 1.00, 95%CI 0.96–1.05; p = 0.98)	When adjusted for self-efficacy towards adherence to medications, no difference (OR = 1.00, 95%CI 0.95–1.06; p = 0.85)	Subjective measures of adherence; adolescents may have been less likely to take ART due to being healthier and exhibiting fewer symptoms
Health Literacy and Demographic Disparities in HIV Care Continuum Outcomes (2018)	Rebeiro et al	575 adults in southern US	Health literacy measured via BHLS done by clinician ART adherence measured via questionnaire (missed ART in a week), and retention in care determined via if patient made 2 + visits 90 days apart in 1 year	No significant association between retention in care and health literacy (aRR = 1.10, 95%CI 0.84–1.43)	No significant association between medication adherence and health literacy (aRR = 1.01, 95%CI 0.73–1.40)		Individuals may have sought care outside of clinic but marked as lack of retention; adherence was by self-report; results may not be generalizable outside of southern US
Health Literacy: An Overlooked Factor in Understanding HIV Health Disparities (2007)	Osborn et al	204 adults from Chicago and Louisiana	Health literacy measured via REALM Medication adherence measured via PMAQ (missed dosages in 4 days)		Health literacy significantly predicted nonadherence (aOR = 2.12, 95% CI 1.93–2.32)	Health literacy mediates the relationship between race and adherence. When literacy factored in, medication adherence differences between white and African-American patients became nonsignificant (aOR = 1.80, 95% CI = 0.51–5.85; C statistic = 0.72)	Could not use objective measures of adherence so missed doses may have been underreported; the data comes from interviews from 5 years prior
Risk and Protective Factors for Retention in HIV Care (2014)	Waldrop-Valverde et al	210 adults from Miami and South Florida	Health Literacy measured by S-TOFHLA Retention in care measured through number of kept visits divided by number of visits scheduled over 28 weeks	No association between health literacy and adherence to medical or phlebotomy visits (test statistic not shown)		Neither patient-provider relationship nor social support was a moderator of the effects of health literacy on phlebotomy visit adherence	Limited geographical region; did not look into clinic factors that affect retention; study population recruited solely from clinic
The Association between Health Literacy and HIV Treatment Adherence: Further Evidence from Objectively Measured (2008)	Kalichman et. al	145 adults in Atlanta, GA	Health literacy measured via TOFHLA HIV treatment adherence measured by monthly unannounced pill counts (pills counted/pills prescribed)		Association between lower health literacy and poorer adherence (OR = 3.77, 95%CI 1.46 – 9.93; p < .01)		Small sample size; narrow geographical region; only used TOFHLA; unmeasured outside factors (e.g. treatment attitudes) may mediate this relationship
Adherence to Combination Antiretroviral Therapies in HIV Patients of Low Health Literacy (1999)	Kalichman et. al	318 adults in Atlanta, GA	Health Literacy measured via TOFHLA Medication adherence measured via questionnaire (included total AR pills taken/total AR pills prescribed)		Association between lower health literacy and HAART nonadherence was significant (OR = 3.9, 95%CI 1.1 – 13.4; p < .05)	When level of education (< 12 years) factored in, relationship between health literacy and adherence became nonsignificant	Self-reported data no assessment of neurocognitive impairment; only one assessment of health literacy; used flyers to recruit which may leave out people with lower literacy
Critical, and not functional, health literacy is associated with missed HIV clinic visits in adults and older adults living with HIV in the Deep South (2020)	Fazeli et al	95 adults from Southeastern US clinic	Health literacy measured by several areas (reading, self-efficacy, numeracy, and ability to appraise/access health info). Capacity to appraise/access health info specifically measured through NVS Engagement in care measured through number of missed visits/number of scheduled visits	Association found between health literacy and retention. Ability to appraise health info, specifically, was strong predictor of missed visits (β = -0.46, [-8.71]–[-1.86]; p < 0.001)		Greater levels of depressive symptoms, poorer neurocognitive functioning independent predictors of adherence	Used retrospective data for missed clinic visits; not all factors that affect visits accounted for; narrow demographics; small sample size
Predictors of disparities in retention in care among African Americans living with HIV (2020)	Anderson et al	699 adults from Atlanta, Georgia	Health literacy measured via S-TOFHLA Retention in care measured as number of kept visits/number of scheduled visits	Health literacy was the main predictor of 100% visit adherence (OR = 1.02, 95%CI 1.00–1.04; p = 0.024)		Health literacy mediates the relationship between race and adherence. When health literacy factored in, race became nonsignificant factor for retention in care. As patients viewed provider more favorably (measured by Attitudes Towards the HIV Healthcare Provider Scale), visit adherence decreased (β = -0.10, [-0.2]–[-0.01]; p = 0.037)	Not all possible factors accounted for; patients not retained in care not represented well, results may not be generalizable
Literacy, self-efficacy, and HIV medication adherence (2006)	Wolf et al	204 adults from Louisiana and Chicago	Health literacy measured via REALM Medication adherence to antiretroviral regimens measured via PMAQ (missed dosages in 4 days)		Association between low patient health literacy level and medication nonadherence in past 4 days (AOR 3.3, 95% CI 1.3–8.7)	Self-efficacy moderated the impact of low health literacy on medication adherence, reducing the association by 40% (AOR = 7.4, 95% CI 2.7–12.5)	Self-reported data; unable to control for other factors
Literacy, Social Stigma, and HIV Medication Adherence (2008)	Waite et al	204 PWH who received clinical care in Shreveport, Louisiana and Chicago, Illinois	Health literacy measured via REALM Medication adherence measured via PMAQ (missed dosages in 4 days)		Patients with low literacy were more than 3 × more likely to miss dosages in HAART (AOR = 3.3, 95% CI 1.3–8.7; p < 0.001)	Perceived social stigma moderated relationship between low literacy and improper adherence, reducing effect by 40% (AOR 3.1, 95% CI 1.3–7.7)	Did not measure other forms of stigma; self- reported data; results not generalizable from small sample size
Health Literacy, Antiretroviral Adherence, and HIV-RNA Suppression (2006)	Paasche-Orlow et. al	235 PWH with a history of alcohol abuse from Boston	Health literacy measured via REALM ART adherence measured via a questionnaire (if 100% adherent over previous 3 days)		People with lower health literacy had a higher chance of ART adherence (unadjusted OR = 2.23, 95% CI 1.15–4.30)		Self-reported data; adherence measures may not have been suited for people with low literacy; REALM not sufficient test for health literacy, narrow demographics so results not generalizable
Health Literacy and Treatment Adherence in Hispanic HIV-infected Patients (2009)	Alcaide et al	60 Hispanic adult PWH from Miami	Health literacy measured by S-TOFHLA Adherence measured by AACTG adherence interview (any doses missed in past 30 days, number of days without medication in last 4 days)		No relationship between poor health literacy and poor adherence to medication regimen (test statistic not shown)		Small sample size, self-reported data, STOFHLA not comprehensive enough, narrow demographic so results not generalizable, may not have gotten good representation of very low health literacy, potential language barrier problems
Knowledge of Antiretroviral Regimen Dosing and Adherence: A Longitudinal Study (2003)	Miller et al	128 PWH starting a new HAART regimen	Health literacy measured via TOFHLA (Medication knowledge score, or MKS, was calculated as a separate variable Adherence measured by pill count, self-reported adherence (missed dosages in prior week), and MEMS electronic bottle caps		At week 8, bivariate analyses showed low health literacy independently predicted low MKS (correlation coefficient = 0.31, p = 0.005), which was consistent in multivariate model; at week 8, MKS was associated with adherence (test statistic not shown)		9% of data could not be analyzed since they did not complete multiple surveys; self- reported data; small sample size
*Qualitative articles*							
Health Literacy in HIV Treatment: Accurate Understanding of Key Biological Treatment Principles is Not Required for Good ART Adherence (2015)	Laws et. al	32 adults in 2 New England cities	In-depth interviews with patients		Most had very limited biomedical understanding of HIV and antiretroviral therapy, however most reported good. Authors concluded most participants simply followed doctors' instructions without deep understanding		Limited geographical region; no new diagnoses; small sample size; unclear if healthcare providers had educated them on HIV or treatment during study
Mapping Patient–Identified Barriers and Facilitators to Retention in HIV Care and Antiretroviral Therapy Adherence to Andersen's Behavioral Model (2015)	Holtzman et al	51 adults in clinics in Philadelphia, PA	In-depth interviews with participants	Low health literacy was not found to be a barrier to retention	Low health literacy found to be a barrier to adherence	Patients more likely to adhere to medications when reported providers spent time talking to them about their medications; Stigma cited as a barrier to retention; social support cited as a facilitator of both adherence and retention	Only looked at patients in primary HIV care; small sample size; sample mainly included heterosexual racial/ethnic minorities from urban settings; self-reported data
Building Trust and Relationships Between Patients and Providers: An Essential Complement to Health Literacy in HIV Care (2016)	Dawson-Rose et al	28 focus groups with adult patients (135) and providers (71) from all over US and territories	PWH participants completed survey and participated in focus groups	Patients felt health literacy tied to trust in provider which determines retention in care	Patients felt health literacy tied to trust in provider which determines ART adherence	Long term patient-provider relationship with trust deemed critical to association between health literacy and adherence/retention. Minority participants had lower trust in health system	Questions focused more on the process of informational gathering; Participants were solely from clinics/HIV organizations

## Quantitative Instruments & Measures

### Health Literacy

Health literacy was assessed by different measures throughout the literature, although the majority used some form of either the TOFHLA or the REALM. Four studies used the TOFHLA and an additional three used the shortened version (S-TOFHLA). Five studies utilized the REALM. Of those, four used the REALM for adults and one study used the REALM-teen version. One study used brief health literacy screens (BHLS) that measure one’s confidence in filling out medical forms [19]. Another study utilized a health literacy survey that measured reading, self-efficacy, numeracy, and ability to appraise and/or access health info, and that same study also used the Newest Vital Sign questionnaire (NVS), which assesses how well a patient can evaluate and comprehend health information [20].

### ART Adherence

ART adherence measures varied from self-reported questionnaires (including the Patient Medication Adherence Questionnaire, or PMAQ, Adult AIDS Clinical Trials Group adherence interview, or AACTG, and the Pediatric Adherence Questionnaire) to unannounced monthly pill counts, or MEMS electronic bottle caps, which record the number of times the pill bottles were opened.

### Retention in Care

Four studies examined retention in care through medical chart abstraction. In three studies, retention in care was measured as the proportion of kept visits or missed visits out of all scheduled visits, while one study used the Health and Resources Services Administration HIV/AIDS Bureau (HRSA HAB) definition defined by whether a patient had two kept visits separated by > 90 days during the 12-month observation period [18].

### Qualitative Studies

Two qualitative studies involved in-depth one-on-one interviews, and one involved focus group interviews with patients, providers, and professional care team members (i.e., social workers, physician assistants, nurses) [21–23]. Questions included HIV knowledge of the consequences of non-adherence, barriers or facilitators to either retention or adherence, and where and how patients were diagnosed with HIV. Assessment of health literacy was done primarily through open-ended questions that tested patient knowledge on basic medical facts, including questions about HIV science and antiretroviral therapy, as well as participants’ ability to follow medication regimens. For example, one study used an explanatory model of questioning to assess health literacy, by asking participants, “If you were going to explain to a friend what HIV is, what would you say?” [21] Another study used the Andersen’s Behavioral Model to assess barriers and facilitators to ART adherence and retention in care, and coded excerpts from interviews associated with health literacy. Although these approaches differed from the quantitative assessments of health literacy and they focused solely on HIV, they similarly assessed the components of health literacy aforementioned in the Introduction – basic knowledge and understanding of concepts related to HIV, as well as the ability to follow a medication regimen.

ART adherence and retention in care were both explored through questions in the interviews about general adherence or retention in all of the studies. In one study, while health literacy was measured qualitatively, ART adherence was measured quantitatively using pill counts, MEMS, and self-report [24].

### The Association Between Health Literacy and Medication Adherence

Table 1 includes a comprehensive list of all studies. Among the 11 quantitative studies exploring the association between health literacy and medication adherence, 64% (n = 7) found a statistically significant association [24–30]. In these studies, PWH with lower levels of health literacy were less likely to take their ART correctly or at all. Both Waite et al. and Wolf et al., using the same measure of health literacy (REALM) and of ART adherence (PMAQ), found that PWH with lower levels of health literacy were more than three times as likely to miss their medication doses [27, 28]. In a longitudinal study, Miller et al. examined the association of medication knowledge, health literacy and adherence, using 1) a Medication Knowledge questionnaire with items on names of medications, number of doses per day for each medicine, and number of pills for each dose 2) the TOFLHA to assess health literacy, and 3) three measures of adherence (self-report, pill count and MEMS) [24]. In a multivariable model, they found that low health literacy independently predicted their Medication Knowledge Score at week 8 (correlation coefficient = 0.31, p = 0.005), which in turn was associated with lower ART adherence [24]. However, after week 8, Medication Knowledge scores plateaued and adherence remained less than adequate (less than 85%). The authors concluded medication knowledge was critical but not sufficient to achieve optimal adherence [24].

Rebeiro et al., using BHLS to measure health literacy, found that poor health literacy was associated with lower rates of viral suppression [19]. However, the study did not find a significant association between health literacy and ART adherence, as measured by self-report (aRR = 1.01, 95%CI 0.73–1.40) [19]. Navarra et al., in a study of adolescent PWH (age 13 through 24) also did not find a significant association between health literacy and ART adherence, with health literacy measured using the TOFHLA and REALM-teen, and ART adherence measured by self-report (OR = 0.954, 95%CI 0.893–1.018; p = 0.15) [31]. Likewise, when Murphy et al. studied PWH between the ages of 16 and 24, no significant association was found between health literacy, measured by TOFHLA, and ART adherence, measured by the Pediatric Adherence Questionnaire (OR = 1.00, 95%CI 0.96–1.05; p = 0.98) [32].

In a counterintuitive finding, Paasche-Orlow et al. found in a longitudinal cohort that poor health literacy (assessed with the REALM) was a predictor of increased ART adherence among PWH who had a history of alcohol use disorder (OR = 2.23, 95% CI 1.15–4.30), while high health literacy predicted decreased ART adherence as measured by self-report among this same population [30].

Among qualitative studies, Holtzman et al. conducted in-depth interviews with PWH, and identified low health literacy (defined as a lack of understanding of how ART biologically fights against HIV) as a barrier to ART adherence [22]. Patients reported that they did not know whether to take their medication with food, whether it was safe to take their pill at a different time each day, or whether they could drink alcohol and take their pills. PWH who did not understand how ART worked physiologically often skipped doses to avoid perceived potential harmful side effects [22]. Laws et al. conducted in-depth interviews of 32 PWH to examine their understanding of HIV science and reasons for ART adherence. Only two out of 32 participants possessed a more nuanced understanding of the disease and treatment, and most had only very limited knowledge. However, most reported good medication adherence potentially due to faith in their doctors and thus, willingness to follow a prescribed treatment regimen even without understanding the science of how ART affects the virus [21].

### The Association Between Health Literacy and Retention in Care

Two out of four quantitative studies that examined retention in care found a significant association between health literacy and retention in HIV care, with PWH with high health literacy levels more likely to attend follow up medical appointments [13, 20]. In a multivariable model, Anderson et al. identified health literacy, measured by S-TOFHLA, as a significant predictor of retention in care, measured as 100% visit adherence (OR = 1.02, 95%CI 1.00–1.04; p = 0.024) [13]. Fazeli et al. found health literacy as measured by the NVS, particularly the ability to appraise health information, was associated with retention in HIV care, defined as proportion of clinic visits in the prior 24 months where the participant did not attend without canceling or rescheduling (β = -0.46, [-8.71]–[-1.86]; p < 0.001) [20].

The other two quantitative studies, however, found that health literacy was not associated with retention in HIV care [19, 33]. Waldrop-Valverde et al. studied health literacy using S-TOFHLA and its relationship to HIV medical care and phlebotomy visit adherence (e.g., number of visits kept divided by number of visits scheduled) with no significant association found [33]. Rebeiro et al., who studied PWH living in the southern U.S. and measured health literacy using BHLS, also found low health literacy was not associated with retention in HIV clinical care (aRR = 1.10, 95%CI 0.84–1.43) [19]. Similarly, in the qualitative study by Holtzman et al., participants did not identify lack of health literacy as a significant barrier to retention in HIV care [22].

### Factors Associated with Health Literacy, ART Adherence and Retention in Care

#### Race/Ethnicity

Four studies examined how health literacy affects the HIV care of PWH of different races/ethnicities. Two studies found evidence that health literacy levels mediate the relationship between race and ART adherence/retention in care. Osborn et al. found white patients were more than twice as likely as Black patients to adhere to their HIV medications. However, when health literacy, as measured by REALM was included in the multivariable regression model, this finding became non-significant (aOR = 1.80, 95% CI = 0.51–5.85; C statistic = 0.72) [29]. Anderson et al. found that non-Black PWH (primarily white patients) were more likely than Black PWH to go to 100% of doctor visits and appointments, controlling for sex, marital status, and sexual orientation. When health literacy was included in the multivariable regression model, racial differences were no longer significant, suggesting that racial differences were likely due to the mediating impact of health literacy, rather than race alone [13]. However, Fazeli et al. found race was not a significant independent correlate of adherence, nor did it mediate the relationship between health literacy and adherence [20]. Similarly, in a study by Alcaide et al. of only Hispanic PWH, no association between health literacy and adherence was found [34].

#### Stigma

One quantitative study explored how HIV-related stigma has been an important barrier to health outcomes in PWH. Waite et al. found that increased experience of HIV-related stigma, assessed using three items from the PMAQ (e.g., “I don’t want people to see me take my HIV medicines”), was directly associated with worse ART adherence and retention in care. They also found that HIV-related perceived stigma partially mediated the relationship between health literacy and medication adherence, reducing the association of health literacy and medication adherence by 40 percent (AOR 3.1, 95% CI 1.3–7.7) [27]. In this study, HIV-related stigma had more of a direct impact on whether patients adhere to ART rather than low health literacy levels. Furthermore in this study, internalized or perceived (actions/remarks not outwardly discriminatory but that can be perceived as such) HIV-related stigma showed a greater moderating effect on medication adherence than enacted (outwardly discriminatory remarks or actions due to diagnosis) stigma [27].

#### Self-Efficacy

Self-efficacy, the belief or confidence in oneself to properly take medications, plays a role in the relationship between health literacy and retention to HIV care or ART adherence. Three studies explored this relationship. Two studies found significant evidence that self-efficacy is associated with health literacy and influences retention and ART adherence. Wolf et al. found poorer health literacy levels led to worse HIV medication adherence; however, when controlling for self-efficacy to take medications, the health literacy effect on adherence dropped by about 40 percent (AOR = 7.4, 95% CI 2.7–12.5) [28]. This implies that self-efficacy moderates this relationship, and is the more salient factor in determining ART adherence rather than health literacy level. Furthermore, Navarra et al., found that those with increased positive outcomes expectancy, or beliefs perceived by an individual on the likelihood of a positive outcome, which is closely related to self-efficacy, were more likely to have 100% adherence to medications and more likely to attend all of their doctor visits (OR = 1.07, 95%CI 1.02–1.12;p = 0.006) [31]. However, Murphy et al., after adjusting for self-efficacy towards medication adherence, found no significant association between health literacy and adherence to ART (OR = 1.00, 95%CI 0.95–1.06; p = 0.85) [32].

### Patient Trust in Provider

Trust was identified as an integral part of the patient-provider relationship in HIV care and may be associated with health literacy, and impact HIV care outcomes. One qualitative study examined whether patient trust in their provider affected the relationship between health literacy and HIV-related health behaviors. Dawson-Rose et al. conducted 28 focus groups and examined aspects of care that influence health literacy and adherence. Trust between patient and providers built over time was one of the most important aspects of care described by the participants [23]. Patients reported that having a trusting relationship with their provider improved their health literacy, their HIV care engagement, and their adherence to HIV medications [23]. Dawson-Rose et al. also found racial and ethnic minority patients living with HIV often expressed less trust in their HIV care providers and the healthcare system as a whole [23]. Some patients were affected by anticipatory stigma related to low health literacy levels. In other words, they feared being shamed for lacking knowledge about HIV. Because of this, the authors posited it may be that they did not trust their provider enough to disclose that they did not understand information about their HIV care and treatment [23].

## Discussion

In spite of tremendous advances in HIV treatment and care, non-adherence and failed viral suppression remain major barriers to ending the HIV epidemic in the US. This systematic review examines not only more recent studies on the associations between health literacy and adherence to treatment and care, but also begins to evaluate studies that identify important psychosocial variables associated with health literacy and its relationship to HIV treatment and care. These variables included HIV-related stigma, self-efficacy, and patient-provider trust, however there are certainly many other potential variables that warrant further exploration. In many aspects of HIV clinical care, there has been a shift from focusing on individual behavioral modifications to addressing the important systemic changes needed in healthcare delivery to achieve greater equity for PWH and thus better health outcomes. In the area of health literacy, there has been a historical focus on more “patient-friendly” ways to present complex HIV medical information, and other modes of assistance like pill boxes, visual charts, greater use of technology, and more robust care coordination for patients with low health literacy [7]. Although important, this approach to health literacy improvement, particularly among racial and ethnic minorities, often does not include factors like those identified in this review that may be associated with health literacy and race, and may be important to improve HIV clinical practice in diverse racial and ethnic populations.

Furthermore, in terms of studies in this review focused on racial and ethnic groups, for half the studies, when health literacy was incorporated into statistical models, there was no longer an association between race and poor HIV outcomes. This finding suggests the need to consider the other correlates of race (e.g., structural) that may be associated with health literacy, beyond socioeconomic status, healthcare access, and education level. While multiple studies examined race as a sociodemographic variable in the context of health literacy, none of the studies in this review examined structural racism. The Centers for Disease Control has re-categorized health literacy into *personal* health literacy and *organizational* health literacy, prompted by studies showing the critical importance of healthcare systems providing access to health information and services equitably [35]. Santana et al. underscores that a shift in focus to organizational health literacy in the context of the HIV epidemic may be vital for improved health outcomes [35].

One’s race and experiences with systemic racism in HIV care affects level of trust in providers [36]. Our review found that studies have shown a patient’s level of trust in their provider affected both their health literacy and their HIV outcomes. Patients must receive HIV medical information from a trusted source, which may be the physician, but may also be the nurse, social worker or peer educator [37]. Finding ways to make healthcare providers and institutions more trustworthy, rather than focusing solely on increasing the patient’s level of trust, may bolster the chances that patients receive and process medical information successfully.

Our review also found that internalized or perceived HIV-related stigma affects how PWH receive and integrate information about their HIV care. While few studies examined stigma, there is emerging literature pointing to stigma as a potentially important mediator or moderator that is addressable in the context of health literacy, and how HIV-related stigma and stigma related to one’s level of health literacy may reinforce each other. Addressing how structural HIV stigma (e.g. the policies and norms of a clinical site) as well as provider attitudes and behaviors perpetuate internalized or perceived HIV-related stigma among their patient population, which in turn may silence PWH who have low health literacy, may be critical for better HIV outcomes [38]. Health literacy and its relationship with other forms of structural oppression (e.g., racism, homophobia, transphobia) warrant further elucidation in the context of HIV stigma through an intersectional lens.

Self-efficacy was also shown to moderate the impact of low health literacy on HIV-related health behaviors in our review. It may be important to investigate how low health literacy within HIV care may lead to patients being unable to ask the right questions to understand their treatment. Katz et al. found, in general, patients with increased health literacy were more likely to ask about any concerns related to managing and treating their condition [39, 40]. Patients with lower levels of health literacy may not know the right questions to ask, nor feel comfortable asking, to comprehend their diagnosis and/or treatment and then do not learn how to follow their medication regime correctly, facing potential adverse effects. This dynamic should be studied specifically within HIV care, to see if this plays a role in the development of self-efficacy in achieving favorable HIV care outcomes.

This review elucidates some potentially key psychosocial variables, such as race, stigma, self-efficacy, and patient-provider trust, that may not only independently be associated with one’s level of health literacy, but may also be associated with each other as well. For example, a patient may not trust their provider if they fear being stigmatized for having poor health literacy [23]. If PWH are shamed or anticipate HIV- or health literacy-related stigma, they may feel less confident or exhibit lower levels of self-efficacy [41, 42]. HIV- and health literacy-related stigma, trust in providers, and self-efficacy can all be affected by one’s experience with structural racism in healthcare, including from their providers. Previous experiences with racism can affect patients’ level of trust in the patient-provider relationship, particularly for Black patients, given a potential association of a provider with a system that perpetuates experienced racism [23, 43]. Few studies identified here examined the mediating, moderating, or confounding effects of the potentially complex behavioral relationships between these variables, when exploring the causal relationship between health literacy and HIV outcomes. This review provides an avenue for further exploration of health literacy as a persistent, and critical variable in determining how patients interact within a complex HIV healthcare system.

### Limitations

There were a few limitations to this systematic review. Studies included in this review were limited to the U.S. There are likely other important psychosocial variables from global studies that may contribute to our understanding of health literacy and the HIV epidemic in the U.S. Furthermore, while including both qualitative and quantitative studies add to a comprehensive picture of health literacy in HIV, it also means a lack of a common standard across different methodological approaches. Only half the studies found an association between health literacy and ART adherence or retention in care, which may have been due to many factors, including how health literacy was defined and measured within each study and which component of the health literacy construct was emphasized. For example, when studies used TOFHLA or S-TOFHLA to measure health literacy, which emphasize basic skills with numbers and reading, about the same number of studies did and did not find an association between health literacy and adherence. Similarly, when studies used the REALM test or REALM-teen, which solely focuses on reading ability of health-related vocabulary, three out of the five studies found low health literacy to predict ART nonadherence. One found it did not predict adherence, and the other found low health literacy to predict high ART adherence [30, 32]. Another key element of health literacy–knowledge of one’s medication regime–did not predict adherence or care retention [24], yet the capacity to appraise health information was associated with improved retention in care [20]. Finally, many studies used self-report methods for assessment of adherence. Self-report can overestimate adherence due to social desirability bias and memory issues. More consistent use of health literacy measures or constructs, as well as more reliable measures of ART adherence (e.g. blood drug levels) may lead to more consistent and valid findings.

### Future Directions

In addition to the psychosocial variables presented in this review, other factors related to structural racism, such as experiences of discrimination and racialized trauma may play important roles in health disparities and ultimately HIV health outcomes. Thus, in addition to learning how to support the health literacy of patients, helping providers identify and understand factors such as racialized trauma and their role in influencing adherence and care may be equally important. It is equally important for HIV care providers to have high levels of literacy around racialized trauma and the lived experiences of PWH and their own potential implicit racial biases, as it is for patients to understand the intricacies of their ART regimen.

## Conclusion

Although the literature is limited, we did find that along with race, the factors of HIV-related stigma, self-efficacy, and patient trust towards providers are associated with health literacy in some studies, and they may have a mediating or moderating effect on ART adherence and retention in HIV care. Clearly, more studies with larger populations are needed. Yet, these factors likely provide a ripe area for further study and novel interventions that address the challenges and important context when patients enter HIV care and are deemed to have low levels of health literacy. A more comprehensive and consistent methodology to capture health literacy, as well as a more nuanced examination of the influential psychosocial and systemic factors at play, may help to improve the clinical outcomes of PWH, especially among the most vulnerable populations. Ultimately, the need to determine possible factors at play in patients with low health literacy is important to develop effective, equitable health literacy interventions for PWH and enhanced trainings for providers. Future interventions that improve health literacy and address systemic forms of oppression, as well as trustworthiness of institutions and providers, may be critical in addressing non-adherence, and poor health outcomes, particularly in communities of color.

## Data Availability

Not applicable.
